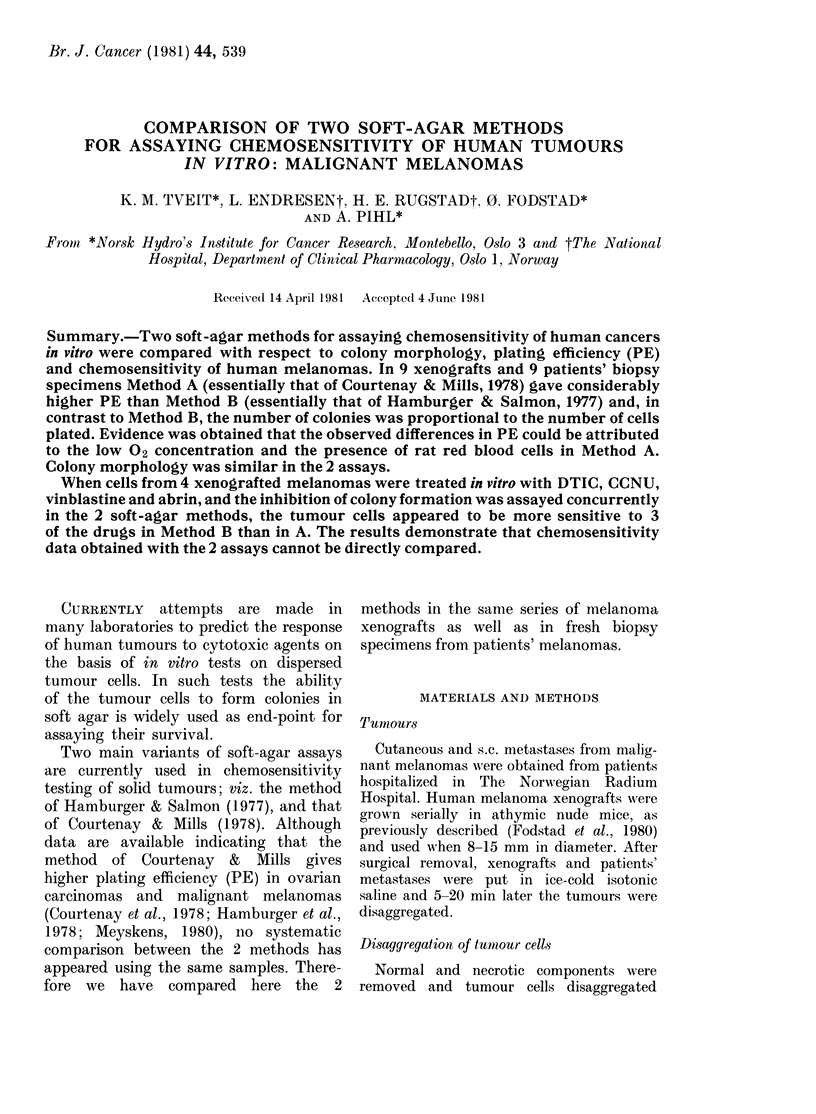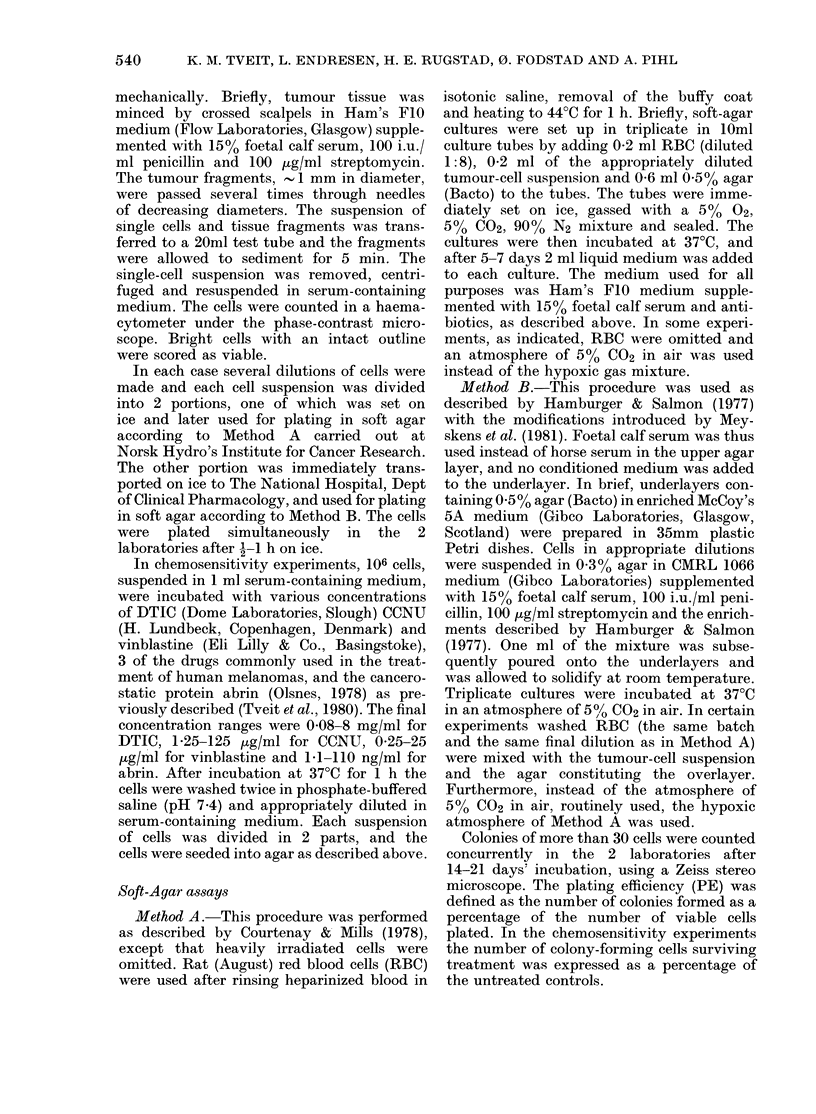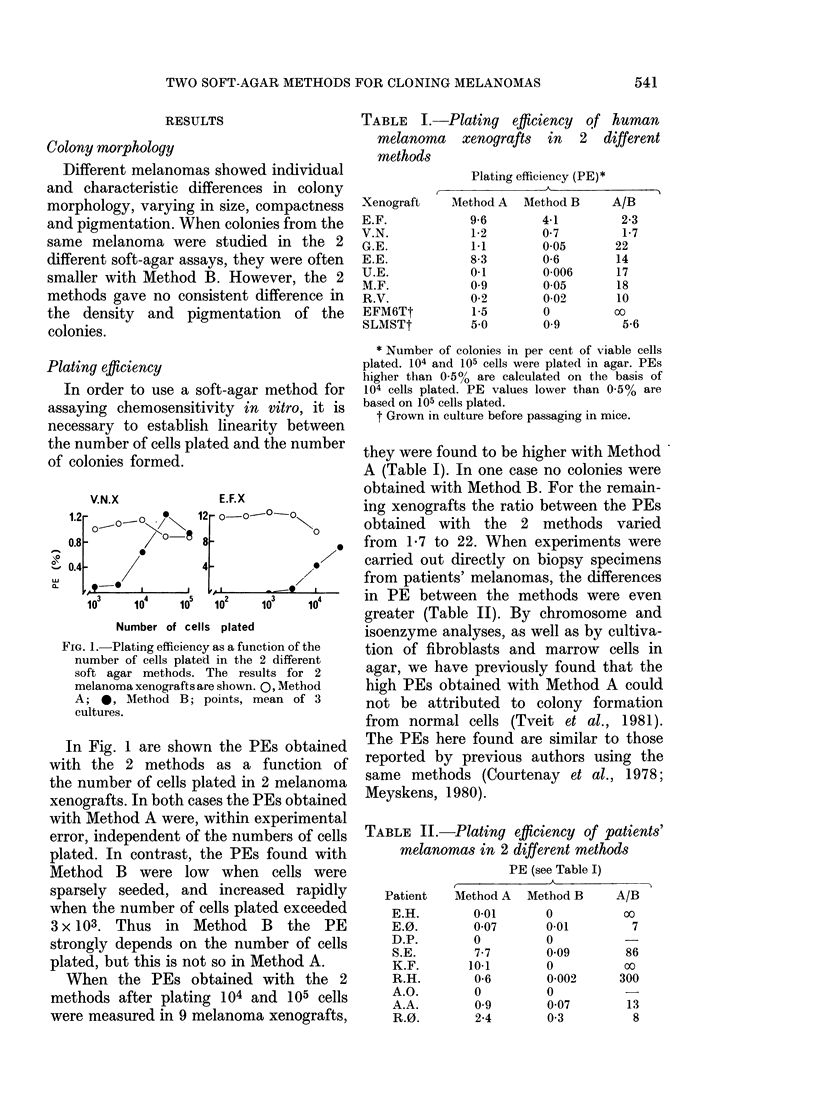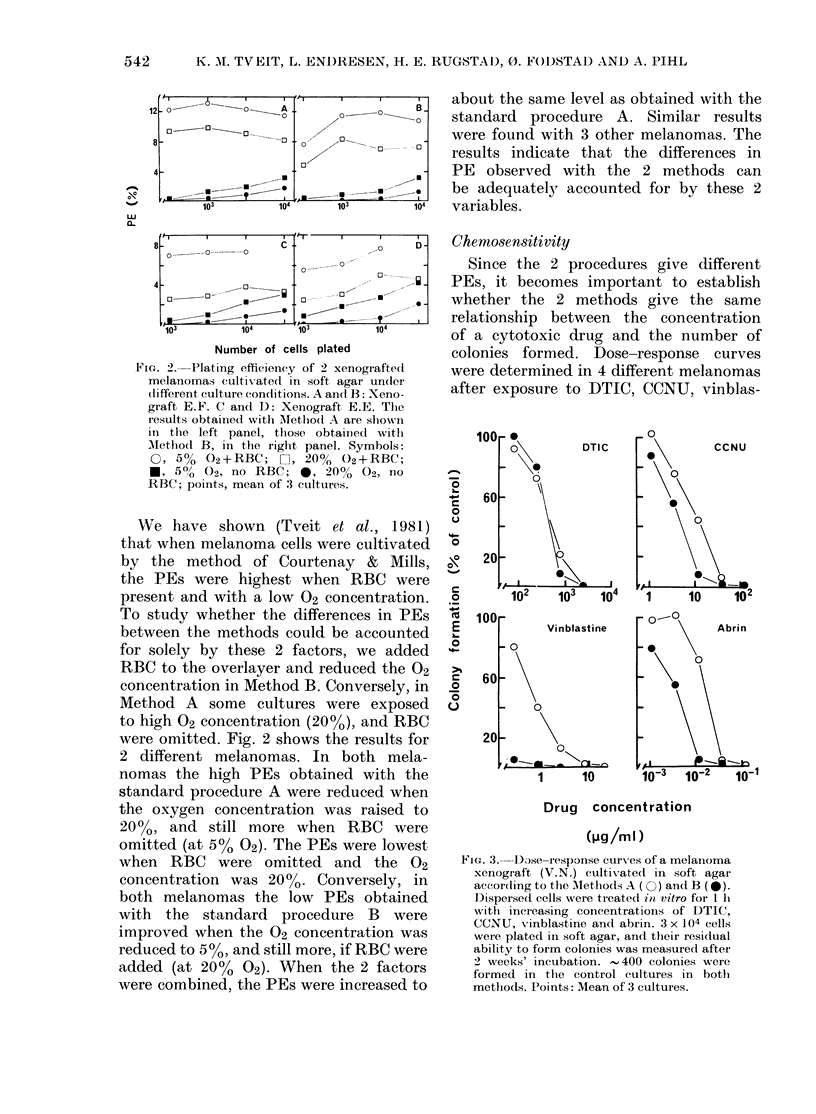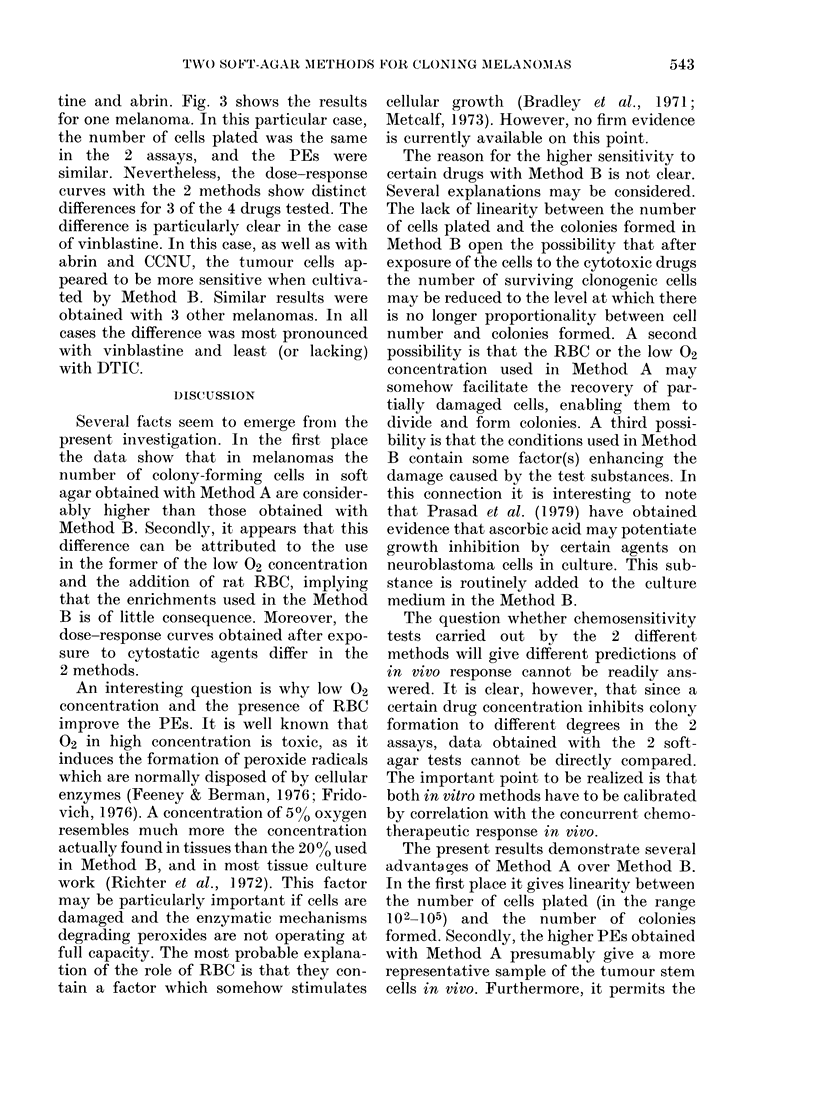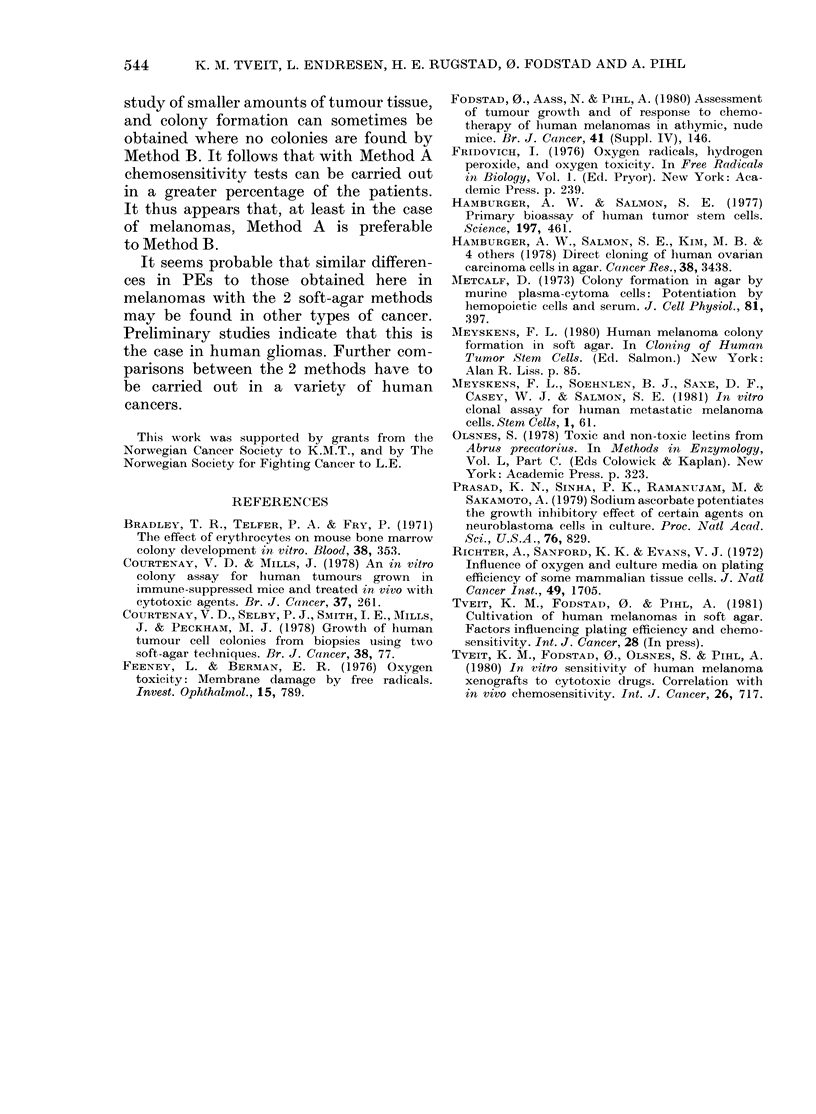# Comparison of two soft-agar methods for assaying chemosensitivity of human tumours in vitro: malignant melanomas.

**DOI:** 10.1038/bjc.1981.223

**Published:** 1981-10

**Authors:** K. M. Tveit, L. Endresen, H. E. Rugstad, O. Fodstad, A. Pihl

## Abstract

Two soft-agar methods for assaying chemosensitivity of human cancers in vitro were compared with respect to colony morphology, plating efficiency (PE) and chemosensitivity of human melanomas. In 9 xenografts and 9 patients' biopsy specimens Method A (essentially that of Courtenay & Mills, 1978) gave considerably higher PE that Method B (essentially that of Hamburger & Salmon, 1977) and, in contrast to Method B, the number of colonies was proportional to the number of cells plated. Evidence was obtained that the observed differences in PE could be attributed to the low O2 concentration and the presence of rat red blood cells in Method A. Colony morphology was similar in the 2 assays. When cells from 4 xenografted melanomas were treated in vitro with DTIC, CCNU, vinblastine and abrin, and the inhibition of colony formation was assayed concurrently in the 2 soft-agar methods, the tumour cells appeared to be more sensitive to 3 of the drugs in Method B than in A. The results demonstrate that chemosensitivity data obtained with the 2 assays cannot be directly compared.


*Br. J. Cancer* (1981) **44**, 539

### COMPARISON OF TWO SOFT-AGAR METHODS FOR ASSAYING CHEMOSENSITIVITY OF HUMAN TUMOURS *IN VITRO*: MALIGNANT MELANOMAS

K. M. TVEIT*, L. ENDRESEN†, H. E. RUGSTAD†, Ø. FODSTAD* AND A. PIHL*

*From *Norsk Hydro's Institute for Cancer Research, Montebello, Oslo 3 and †The National Hospital, Department of Clinical Pharmacology, Oslo 1, Norway*

Received 14 April 1981 Accepted 4 June 1981

**Summary.—Two soft-agar methods for assaying chemosensitivity of human cancers *in vitro* were compared with respect to colony morphology, plating efficiency (PE) and chemosensitivity of human melanomas. In 9 xenografts and 9 patients' biopsy specimens Method A (essentially that of Courtenay & Mills, 1978) gave considerably higher PE than Method B (essentially that of Hamburger & Salmon, 1977) and, in contrast to Method B, the number of colonies was proportional to the number of cells plated. Evidence was obtained that the observed differences in PE could be attributed to the low $O_2$ concentration and the presence of rat red blood cells in Method A. Colony morphology was similar in the 2 assays.**

**When cells from 4 xenografted melanomas were treated *in vitro* with DTIC, CCNU, vinblastine and abrin, and the inhibition of colony formation was assayed concurrently in the 2 soft-agar methods, the tumour cells appeared to be more sensitive to 3 of the drugs in Method B than in A. The results demonstrate that chemosensitivity data obtained with the 2 assays cannot be directly compared.**

Currently attempts are made in many laboratories to predict the response of human tumours to cytotoxic agents on the basis of *in vitro* tests on dispersed tumour cells. In such tests the ability of the tumour cells to form colonies in soft agar is widely used as end-point for assaying their survival.

Two main variants of soft-agar assays are currently used in chemosensitivity testing of solid tumours; *viz.* the method of Hamburger & Salmon (1977), and that of Courtenay & Mills (1978). Although data are available indicating that the method of Courtenay & Mills gives higher plating efficiency (PE) in ovarian carcinomas and malignant melanomas (Courtenay *et al.*, 1978; Hamburger *et al.*, 1978; Meyskens, 1980), no systematic comparison between the 2 methods has appeared using the same samples. Therefore we have compared here the 2 methods in the same series of melanoma xenografts as well as in fresh biopsy specimens from patients' melanomas.

### MATERIALS AND METHODS

### *Tumours*

Cutaneous and s.c. metastases from malignant melanomas were obtained from patients hospitalized in The Norwegian Radium Hospital. Human melanoma xenografts were grown serially in athymic nude mice, as previously described (Fodstad *et al.*, 1980) and used when 8–15 mm in diameter. After surgical removal, xenografts and patients' metastases were put in ice-cold isotonic saline and 5–20 min later the tumours were disaggregated.

### *Disaggregation of tumour cells*

Normal and necrotic components were removed and tumour cells disaggregated mechanically. Briefly, tumour tissue was minced by crossed scalpels in Ham's F10 medium (Flow Laboratories, Glasgow) supplemented with 15% foetal calf serum, 100 i.u./ml penicillin and 100 μg/ml streptomycin. The tumour fragments, ~1 mm in diameter, were passed several times through needles of decreasing diameters. The suspension of single cells and tissue fragments was transferred to a 20ml test tube and the fragments were allowed to sediment for 5 min. The single-cell suspension was removed, centrifuged and resuspended in serum-containing medium. The cells were counted in a haemacytometer under the phase-contrast microscope. Bright cells with an intact outline were scored as viable.

In each case several dilutions of cells were made and each cell suspension was divided into 2 portions, one of which was set on ice and later used for plating in soft agar according to Method A carried out at Norsk Hydro's Institute for Cancer Research. The other portion was immediately transported on ice to The National Hospital, Dept of Clinical Pharmacology, and used for plating in soft agar according to Method B. The cells were plated simultaneously in the 2 laboratories after ½–1 h on ice.

In chemosensitivity experiments, $10^6$ cells, suspended in 1 ml serum-containing medium, were incubated with various concentrations of DTIC (Dome Laboratories, Slough) CCNU (H. Lundbeck, Copenhagen, Denmark) and vinblastine (Eli Lilly & Co., Basingstoke), 3 of the drugs commonly used in the treatment of human melanomas, and the cancerostatic protein abrin (Olsnes, 1978) as previously described (Tveit *et al.*, 1980). The final concentration ranges were 0·08–8 mg/ml for DTIC, 1·25–125 μg/ml for CCNU, 0·25–25 μg/ml for vinblastine and 1·1–110 ng/ml for abrin. After incubation at 37°C for 1 h the cells were washed twice in phosphate-buffered saline (pH 7·4) and appropriately diluted in serum-containing medium. Each suspension of cells was divided in 2 parts, and the cells were seeded into agar as described above.

### *Soft-Agar assays*

*Method A*.—This procedure was performed as described by Courtenay & Mills (1978), except that heavily irradiated cells were omitted. Rat (August) red blood cells (RBC) were used after rinsing heparinized blood in isotonic saline, removal of the buffy coat and heating to 44°C for 1 h. Briefly, soft-agar cultures were set up in triplicate in 10ml culture tubes by adding 0·2 ml RBC (diluted 1:8), 0·2 ml of the appropriately diluted tumour-cell suspension and 0·6 ml 0·5% agar (Bacto) to the tubes. The tubes were immediately set on ice, gassed with a 5% $O_2$, 5% $CO_2$, 90% $N_2$ mixture and sealed. The cultures were then incubated at 37°C, and after 5–7 days 2 ml liquid medium was added to each culture. The medium used for all purposes was Ham's F10 medium supplemented with 15% foetal calf serum and antibiotics, as described above. In some experiments, as indicated, RBC were omitted and an atmosphere of 5% $CO_2$ in air was used instead of the hypoxic gas mixture.

*Method B*.—This procedure was used as described by Hamburger & Salmon (1977) with the modifications introduced by Meyskens *et al.* (1981). Foetal calf serum was thus used instead of horse serum in the upper agar layer, and no conditioned medium was added to the underlayer. In brief, underlayers containing 0·5% agar (Bacto) in enriched McCoy's 5A medium (Gibco Laboratories, Glasgow, Scotland) were prepared in 35mm plastic Petri dishes. Cells in appropriate dilutions were suspended in 0·3% agar in CMRL 1066 medium (Gibco Laboratories) supplemented with 15% foetal calf serum, 100 i.u./ml penicillin, 100 μg/ml streptomycin and the enrichments described by Hamburger & Salmon (1977). One ml of the mixture was subsequently poured onto the underlayers and was allowed to solidify at room temperature. Triplicate cultures were incubated at 37°C in an atmosphere of 5% $CO_2$ in air. In certain experiments washed RBC (the same batch and the same final dilution as in Method A) were mixed with the tumour-cell suspension and the agar constituting the overlayer. Furthermore, instead of the atmosphere of 5% $CO_2$ in air, routinely used, the hypoxic atmosphere of Method A was used.

Colonies of more than 30 cells were counted concurrently in the 2 laboratories after 14–21 days' incubation, using a Zeiss stereo microscope. The plating efficiency (PE) was defined as the number of colonies formed as a percentage of the number of viable cells plated. In the chemosensitivity experiments the number of colony-forming cells surviving treatment was expressed as a percentage of the untreated controls.

RESULTS

*Colony morphology*

Different melanomas showed individual and characteristic differences in colony morphology, varying in size, compactness and pigmentation. When colonies from the same melanoma were studied in the 2 different soft-agar assays, they were often smaller with Method B. However, the 2 methods gave no consistent difference in the density and pigmentation of the colonies.

*Plating efficiency*

In order to use a soft-agar method for assaying chemosensitivity *in vitro*, it is necessary to establish linearity between the number of cells plated and the number of colonies formed.

FIG. 1.—Plating efficiency as a function of the number of cells plated in the 2 different soft agar methods. The results for 2 melanoma xenografts are shown. ○, Method A; ●, Method B; points, mean of 3 cultures.

In Fig. 1 are shown the PEs obtained with the 2 methods as a function of the number of cells plated in 2 melanoma xenografts. In both cases the PEs obtained with Method A were, within experimental error, independent of the numbers of cells plated. In contrast, the PEs found with Method B were low when cells were sparsely seeded, and increased rapidly when the number of cells plated exceeded $3 \times 10^3$. Thus in Method B the PE strongly depends on the number of cells plated, but this is not so in Method A.

When the PEs obtained with the 2 methods after plating $10^4$ and $10^5$ cells were measured in 9 melanoma xenografts,

TABLE I.—*Plating efficiency of human melanoma xenografts in 2 different methods*

| Xenograft | Plating efficiency (PE)* Method A | Method B | A/B |
|---|---|---|---|
| E.F. | 9·6 | 4·1 | 2·3 |
| V.N. | 1·2 | 0·7 | 1·7 |
| G.E. | 1·1 | 0·05 | 22 |
| E.E. | 8·3 | 0·6 | 14 |
| U.E. | 0·1 | 0·006 | 17 |
| M.F. | 0·9 | 0·05 | 18 |
| R.V. | 0·2 | 0·02 | 10 |
| EFM6T† | 1·5 | 0 | ∞ |
| SLMST† | 5·0 | 0·9 | 5·6 |

* Number of colonies in per cent of viable cells plated. $10^4$ and $10^5$ cells were plated in agar. PEs higher than 0·5% are calculated on the basis of $10^4$ cells plated. PE values lower than 0·5% are based on $10^5$ cells plated.

† Grown in culture before passaging in mice.

they were found to be higher with Method A (Table I). In one case no colonies were obtained with Method B. For the remaining xenografts the ratio between the PEs obtained with the 2 methods varied from 1·7 to 22. When experiments were carried out directly on biopsy specimens from patients' melanomas, the differences in PE between the methods were even greater (Table II). By chromosome and isoenzyme analyses, as well as by cultivation of fibroblasts and marrow cells in agar, we have previously found that the high PEs obtained with Method A could not be attributed to colony formation from normal cells (Tveit *et al.*, 1981). The PEs here found are similar to those reported by previous authors using the same methods (Courtenay *et al.*, 1978; Meyskens, 1980).

TABLE II.—*Plating efficiency of patients' melanomas in 2 different methods*

| Patient | PE (see Table I) Method A | Method B | A/B |
|---|---|---|---|
| E.H. | 0·01 | 0 | ∞ |
| E.Ø. | 0·07 | 0·01 | 7 |
| D.P. | 0 | 0 | — |
| S.E. | 7·7 | 0·09 | 86 |
| K.F. | 10·1 | 0 | ∞ |
| R.H. | 0·6 | 0·002 | 300 |
| A.O. | 0 | 0 | — |
| A.A. | 0·9 | 0·07 | 13 |
| R.Ø. | 2·4 | 0·3 | 8 |

FIG. 2.—Plating efficiency of 2 xenografted melanomas cultivated in soft agar under different culture conditions. A and B: Xenograft E.F. C and D: Xenograft E.E. The results obtained with Method A are shown in the left panel, those obtained with Method B, in the right panel. Symbols: ○, 5% $O_2$+RBC; □, 20% $O_2$+RBC; ■, 5% $O_2$, no RBC; ●, 20% $O_2$, no RBC; points, mean of 3 cultures.

We have shown (Tveit *et al.*, 1981) that when melanoma cells were cultivated by the method of Courtenay & Mills, the PEs were highest when RBC were present and with a low $O_2$ concentration. To study whether the differences in PEs between the methods could be accounted for solely by these 2 factors, we added RBC to the overlayer and reduced the $O_2$ concentration in Method B. Conversely, in Method A some cultures were exposed to high $O_2$ concentration (20%), and RBC were omitted. Fig. 2 shows the results for 2 different melanomas. In both melanomas the high PEs obtained with the standard procedure A were reduced when the oxygen concentration was raised to 20%, and still more when RBC were omitted (at 5% $O_2$). The PEs were lowest when RBC were omitted and the $O_2$ concentration was 20%. Conversely, in both melanomas the low PEs obtained with the standard procedure B were improved when the $O_2$ concentration was reduced to 5%, and still more, if RBC were added (at 20% $O_2$). When the 2 factors were combined, the PEs were increased to about the same level as obtained with the standard procedure A. Similar results were found with 3 other melanomas. The results indicate that the differences in PE observed with the 2 methods can be adequately accounted for by these 2 variables.

### *Chemosensitivity*

Since the 2 procedures give different PEs, it becomes important to establish whether the 2 methods give the same relationship between the concentration of a cytotoxic drug and the number of colonies formed. Dose–response curves were determined in 4 different melanomas after exposure to DTIC, CCNU, vinblas-

FIG. 3.—Dose–response curves of a melanoma xenograft (V.N.) cultivated in soft agar according to the Methods A (○) and B (●). Dispersed cells were treated *in vitro* for 1 h with increasing concentrations of DTIC, CCNU, vinblastine and abrin. $3 \times 10^4$ cells were plated in soft agar, and their residual ability to form colonies was measured after 2 weeks' incubation. ~400 colonies were formed in the control cultures in both methods. Points: Mean of 3 cultures.

tine and abrin. Fig. 3 shows the results for one melanoma. In this particular case, the number of cells plated was the same in the 2 assays, and the PEs were similar. Nevertheless, the dose–response curves with the 2 methods show distinct differences for 3 of the 4 drugs tested. The difference is particularly clear in the case of vinblastine. In this case, as well as with abrin and CCNU, the tumour cells appeared to be more sensitive when cultivated by Method B. Similar results were obtained with 3 other melanomas. In all cases the difference was most pronounced with vinblastine and least (or lacking) with DTIC.

### DISCUSSION

Several facts seem to emerge from the present investigation. In the first place the data show that in melanomas the number of colony-forming cells in soft agar obtained with Method A are considerably higher than those obtained with Method B. Secondly, it appears that this difference can be attributed to the use in the former of the low $O_2$ concentration and the addition of rat RBC, implying that the enrichments used in the Method B is of little consequence. Moreover, the dose–response curves obtained after exposure to cytostatic agents differ in the 2 methods.

An interesting question is why low $O_2$ concentration and the presence of RBC improve the PEs. It is well known that $O_2$ in high concentration is toxic, as it induces the formation of peroxide radicals which are normally disposed of by cellular enzymes (Feeney & Berman, 1976; Fridovich, 1976). A concentration of 5% oxygen resembles much more the concentration actually found in tissues than the 20% used in Method B, and in most tissue culture work (Richter *et al.*, 1972). This factor may be particularly important if cells are damaged and the enzymatic mechanisms degrading peroxides are not operating at full capacity. The most probable explanation of the role of RBC is that they contain a factor which somehow stimulates cellular growth (Bradley *et al.*, 1971; Metcalf, 1973). However, no firm evidence is currently available on this point.

The reason for the higher sensitivity to certain drugs with Method B is not clear. Several explanations may be considered. The lack of linearity between the number of cells plated and the colonies formed in Method B open the possibility that after exposure of the cells to the cytotoxic drugs the number of surviving clonogenic cells may be reduced to the level at which there is no longer proportionality between cell number and colonies formed. A second possibility is that the RBC or the low $O_2$ concentration used in Method A may somehow facilitate the recovery of partially damaged cells, enabling them to divide and form colonies. A third possibility is that the conditions used in Method B contain some factor(s) enhancing the damage caused by the test substances. In this connection it is interesting to note that Prasad *et al.* (1979) have obtained evidence that ascorbic acid may potentiate growth inhibition by certain agents on neuroblastoma cells in culture. This substance is routinely added to the culture medium in the Method B.

The question whether chemosensitivity tests carried out by the 2 different methods will give different predictions of *in vivo* response cannot be readily answered. It is clear, however, that since a certain drug concentration inhibits colony formation to different degrees in the 2 assays, data obtained with the 2 soft-agar tests cannot be directly compared. The important point to be realized is that both *in vitro* methods have to be calibrated by correlation with the concurrent chemotherapeutic response *in vivo*.

The present results demonstrate several advantages of Method A over Method B. In the first place it gives linearity between the number of cells plated (in the range $10^2$–$10^5$) and the number of colonies formed. Secondly, the higher PEs obtained with Method A presumably give a more representative sample of the tumour stem cells *in vivo*. Furthermore, it permits the study of smaller amounts of tumour tissue, and colony formation can sometimes be obtained where no colonies are found by Method B. It follows that with Method A chemosensitivity tests can be carried out in a greater percentage of the patients. It thus appears that, at least in the case of melanomas, Method A is preferable to Method B.

It seems probable that similar differences in PEs to those obtained here in melanomas with the 2 soft-agar methods may be found in other types of cancer. Preliminary studies indicate that this is the case in human gliomas. Further comparisons between the 2 methods have to be carried out in a variety of human cancers.

This work was supported by grants from the Norwegian Cancer Society to K.M.T., and by The Norwegian Society for Fighting Cancer to L.E.

### REFERENCES

BRADLEY, T. R., TELFER, P. A. & FRY, P. (1971) The effect of erythrocytes on mouse bone marrow colony development *in vitro*. *Blood*, **38**, 353.

COURTENAY, V. D. & MILLS, J. (1978) An *in vitro* colony assay for human tumours grown in immune-suppressed mice and treated *in vivo* with cytotoxic agents. *Br. J. Cancer*, **37**, 261.

COURTENAY, V. D., SELBY, P. J., SMITH, I. E., MILLS, J. & PECKHAM, M. J. (1978) Growth of human tumour cell colonies from biopsies using two soft-agar techniques. *Br. J. Cancer*, **38**, 77.

FEENEY, L. & BERMAN, E. R. (1976) Oxygen toxicity: Membrane damage by free radicals. *Invest. Ophthalmol.*, **15**, 789.

FODSTAD, Ø., AASS, N. & PIHL, A. (1980) Assessment of tumour growth and of response to chemotherapy of human melanomas in athymic, nude mice. *Br. J. Cancer*, **41** (Suppl. IV), 146.

FRIDOVICH, I. (1976) Oxygen radicals, hydrogen peroxide, and oxygen toxicity. In *Free Radicals in Biology*, Vol. 1. (Ed. Pryor). New York: Academic Press. p. 239.

HAMBURGER, A. W. & SALMON, S. E. (1977) Primary bioassay of human tumor stem cells. *Science*, **197**, 461.

HAMBURGER, A. W., SALMON, S. E., KIM, M. B. & 4 others (1978) Direct cloning of human ovarian carcinoma cells in agar. *Cancer Res.*, **38**, 3438.

METCALF, D. (1973) Colony formation in agar by murine plasma-cytoma cells: Potentiation by hemopoietic cells and serum. *J. Cell Physiol.*, **81**, 397.

MEYSKENS, F. L. (1980) Human melanoma colony formation in soft agar. In *Cloning of Human Tumor Stem Cells*. (Ed. Salmon.) New York: Alan R. Liss. p. 85.

MEYSKENS, F. L., SOEHNLEN, B. J., SAXE, D. F., CASEY, W. J. & SALMON, S. E. (1981) *In vitro* clonal assay for human metastatic melanoma cells. *Stem Cells*, **1**, 61.

OLSNES, S. (1978) Toxic and non-toxic lectins from *Abrus precatorius*. In *Methods in Enzymology*, Vol. L, Part C. (Eds Colowick & Kaplan). New York: Academic Press. p. 323.

PRASAD, K. N., SINHA, P. K., RAMANUJAM, M. & SAKAMOTO, A. (1979) Sodium ascorbate potentiates the growth inhibitory effect of certain agents on neuroblastoma cells in culture. *Proc. Natl Acad. Sci., U.S.A.*, **76**, 829.

RICHTER, A., SANFORD, K. K. & EVANS, V. J. (1972) Influence of oxygen and culture media on plating efficiency of some mammalian tissue cells. *J. Natl Cancer Inst.*, **49**, 1705.

TVEIT, K. M., FODSTAD, Ø. & PIHL, A. (1981) Cultivation of human melanomas in soft agar. Factors influencing plating efficiency and chemosensitivity. *Int. J. Cancer*, **28** (In press).

TVEIT, K. M., FODSTAD, Ø., OLSNES, S. & PIHL, A. (1980) *In vitro* sensitivity of human melanoma xenografts to cytotoxic drugs. Correlation with *in vivo* chemosensitivity. *Int. J. Cancer*, **26**, 717.